# The Needle in the Haystack: Uncovering the First Whale Shark (*Rhincodon typus*) Aggregation in the Coral Sea

**DOI:** 10.1002/ece3.71552

**Published:** 2025-06-24

**Authors:** Ingo B. Miller, Richard Fitzpatrick, Kátya G. Abrantes, Bradley Norman, Simon J. Pierce, Mark V. Erdmann, Lisa A. Hoopes, Christine Dudgeon, Matthew D. Dunbabin, Alistair D. M. Dove, Robin J. Beaman, Samantha D. Reynolds, Christopher Rohner, Samuel M. Williams, David Paton, Sonny Lewis, Adam Barnett

**Affiliations:** ^1^ Biopixel Oceans Foundation Cairns Queensland Australia; ^2^ AIMS@JCU, College of Science & Engineering James Cook University Townsville Queensland Australia; ^3^ Marine Data Technology Hub James Cook University Townsville Queensland Australia; ^4^ ECOCEAN Inc. Coogee Western Australia Australia; ^5^ Harry Butler Institute Murdoch University Murdoch Western Australia Australia; ^6^ Marine Megafauna Foundation West Palm Beach Florida USA; ^7^ School of Science, Technology and Engineering University of the Sunshine Coast Sunshine Coast Queensland Australia; ^8^ Conservation International Aotearoa University of Auckland Auckland New Zealand; ^9^ Georgia Aquarium Atlanta Georgia USA; ^10^ Robotics and Autonomous Systems, Institute for Future Environments Queensland University of Technology Brisbane Queensland Australia; ^11^ Museum of Science and History Jacksonville Florida USA; ^12^ College of Science and Engineering James Cook University Cairns Queensland Australia; ^13^ Queensland Department of Agriculture and Fisheries Brisbane Queensland Australia; ^14^ The School of Biological Sciences The University of Queensland St Lucia Queensland Australia; ^15^ Blue Planet Marine Canberra, Kingston Australian Capital Territory Australia

**Keywords:** conservation, constellation, elasmobranch, Great Barrier Reef, IUCN, marine megafauna, *Rhincodon typus*

## Abstract

Aggregations are key events, supporting critical ecological and biological functions in many species. For highly mobile and elusive species, aggregations often provide the only feasible opportunities for research. Whale sharks ( *Rhincodon typus* ) form at least 30 consistent seasonal aggregation sites globally, yet none have been documented in the Coral Sea, despite sporadic sightings of solitary individuals and groups. This study aimed to identify and characterise the first whale shark aggregation on Australia's east coast by predicting potential sites through a data layering approach and confirming their presence through targeted field expeditions. A combination of historical sightings data, expert and anecdotal knowledge, and scientific knowledge from other whale shark aggregation sites led to the identification of Wreck Bay, situated at the far northern Great Barrier Reef, as potential aggregation habitat. An initial field expedition in 2019 confirmed the aggregation, and three subsequent voyages in 2021–2024 gathered further demographic and movement data. A total of 59 individuals were identified, with a strong male bias (3.5:1) and all classified as immature sharks ranging from 3.5 to 8.0 m in estimated total length. Satellite tracking revealed a mean residence time of approximately 3 weeks (21.6 days ±10.1 SD; range: 7–43 days), with some individuals revisiting the aggregation in subsequent years. The peak aggregation period occurs from late November to late December, with movements concentrated along the continental shelf before dispersing into the Coral Sea. Tracked sharks (*n* = 18) exhibited wide‐ranging movements, with a mean track duration of 144 days (range: 3–770 days) and a mean total track length of 1463 km (range: 19–11,355 km). This study provides the first evidence of a whale shark aggregation in the Coral Sea and highlights Wreck Bay as key habitat for this iconic and globally endangered species.

## Graphical abstract

This study provides the first documented evidence of a whale shark ( *Rhincodon typus* ) aggregation in the Coral Sea, located at Wreck Bay on Australia's far northern Great Barrier Reef. The site was identified through a combination of historical records and expert input, and confirmed through dedicated field expeditions. The study highlights Wreck Bay as an important habitat for this endangered species and a priority area for future research.

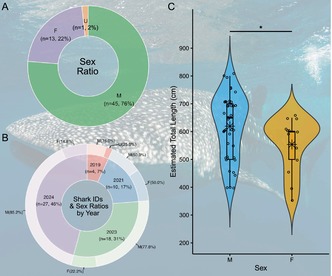

## 1 Introduction

Aggregations are key ecological phenomena observed across a broad spectrum of species of all sizes, both in aquatic and terrestrial environments (Krause and Ruxton 2002; Parrish and Edelstein‐Keshet 1999). These congregations, often of seasonal occurrence, may comprise from tens to millions of individuals, and may include single or multiple species (Parrish and Edelstein‐Keshet 1999). The locations where animals aggregate serve as essential habitats (Barnett et al. 2019; De Wysiecki et al. 2023), playing a crucial role in supporting key biological and ecological functions, including feeding, reproduction and/or shelter (Morrell and James 2008; Parrish and Edelstein‐Keshet 1999). Consequently, these habitats are fundamental to the survival and success of the species that depend on them. For instance, over one million wildebeest ( *Connochaetes taurinus* ) migrate across the Serengeti in East Africa in pursuit of high‐quality vegetation that shifts seasonally (Morrison and Bolger 2012; Subalusky et al. 2017). One of the largest known terrestrial aggregations occurs among straw‐coloured fruit bats ( *Eidolon helvum* ), where up to 10 million individuals gather in Zambia's Kasanka National Park to exploit seasonal food surges (Fahr et al. 2015).

In the marine realm, aggregations often form near oceanographic features such as nutrient‐rich upwelling zones, which sustain high density primary production leading to abundant zooplankton, in turn attracting larger predatory teleosts, sharks, and marine mammals (Botsford et al. 2003; Jacox and Edwards 2011; Johnson et al. 2018; Kingsford and Wolanski 2019). Seasonal events like the South African sardine run or Pacific salmon migration in the Gulf of Alaska exemplify how aggregations can drive predator–prey interactions (Furey et al. 2018; O'Donoghue et al. 2010). Marine aggregations can also serve crucial physiological and reproductive functions. Emperor penguins ( *Aptenodytes forsteri* ), for example, aggregate in large colonies to thermoregulate and enhance breeding success in the Antarctic winter (Ancel et al. 2015), while millions of Christmas Island red crabs (*Gecarcoidea natalis*) conduct synchronised mass migrations to coastal areas for reproduction (Adamczewska and Morris 2001).

Aggregations often present the only feasible opportunity to study certain taxa, particularly for highly mobile and elusive marine species, often due to the logistical challenges and costs involved. A prime example of such species is the whale shark ( *Rhincodon typus* ), the world's largest extant fish, which is classified as Endangered and in decline globally (Pierce and Norman 2016). There are currently approximately 30 known whale shark aggregations, also known as ‘constellations’, across their tropical and subtropical global distribution (Norman et al. 2017; Rohner, Norman, et al. 2021). Whale sharks often aggregate to feed on seasonally available dense patches of zooplankton—their primary food source (Rohner and Prebble 2021). Research at these aggregation sites has been instrumental in advancing scientific understanding of the species, assessing population sizes, informing IUCN Red and Green List evaluations, and guiding conservation efforts (Pierce, Grace, et al. 2021; Pierce and Norman 2016; Rowat and Brooks 2012).

**FIGURE A1:**
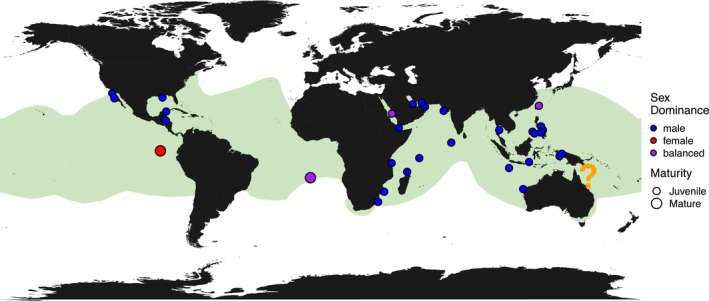
Global distribution of whale shark ( *Rhincodon typus* ) hotspots, with colours indicating sex‐dominance and circle size representing the dominant maturity status at each site. The notable gap in the southwest Pacific region east of Australia across to Polynesia is marked with a ‘?’. The shaded green area represents the species' range distribution, adapted from the IUCN Red List assessment (Pierce and Norman 2016).

The southwest Pacific (SWP) region east of Australia across to Polynesia is a notable blind spot in global scientific data collection on whale shark ecology and conservation (cf. Figure A1; Pierce and Norman 2016; Rohner, Norman, et al. 2021; Womersley et al. 2024). While whale sharks are sporadically sighted along the east coast of Australia and in the Coral Sea, encounters typically involve solitary individuals. Without spatial and temporal knowledge of an aggregation, finding whale sharks in this vast oceanic expanse is akin to finding a needle in a haystack. Given the sporadic sightings, the aim of this study was to determine whether a previously undocumented whale shark aggregation exists on the east coast of Australia and to assess the ecological role and significance of the area to the species, providing a foundation for future research in the region. For this, the study takes two approaches: first, a data layering approach was used to predict the most likely locations for whale shark aggregations to occur (Background Investigation). Second, we conducted field expeditions to confirm the presence of an aggregation at the predicted area, and to characterise the aggregation using demographic information and satellite telemetry.

## 2 Background Investigation: Data Layering Approach

To search for potential whale shark aggregation areas along the expansive eastern Australian coastline with limited resources, we first collated information from a range of sources to narrow down the most promising search areas. These preliminary investigations provided the foundation for the subsequent field expeditions.

### 2.1 Historical Sightings

We first gathered historical records from public databases that report marine species sightings. These included *Sharkbook: Wildbook for Sharks* (https://www.sharkbook.ai) and the *Eye on the Reef* sightings network (https://eotr.gbrmpa.gov.au/home). To complement these, we monitored social media platforms (i.e., Instagram, YouTube, Facebook) for unreported sightings by manually searching terms such as ‘whale shark’ and ‘Great Barrier Reef’. Additionally, we considered unsolicited anecdotal sightings reports provided by members of the public, including fishers and tourism operators. As these were unsolicited and not part of a structural interview process, they did not require ethical approval and were treated as part of the preliminary information‐gathering phase of the study. They were not included in formal analyses, nor did they contribute directly to the main findings. Encounter reports were screened for duplicates, retaining the records with the most detailed information. In cases where the number of animals was reported as a range, or when the numbers differed for the same encounter reported in multiple databases, the midpoint was selected as a conservative estimate. Sightings with no date or adequate location information were omitted. For instance, marlin fishers frequently report encountering whale sharks during the marlin fishing season (October to November); however, only sightings with documented locations were included in the analysis. Additionally, we reviewed the scientific and grey literature to screen for information that might provide insights into historical sightings or ecological patterns associated with whale sharks in the region. While not a formal systematic literature search, we used academic databases including the Web of Science Core Collection (https://www.webofscience.com), Scopus (https://www.scopus.com) and Google Scholar (https://scholar.google.com) search engines with combinations of terms such as ‘whale shark’, ‘ *Rhincodon typus* ’, ‘megafauna’, ‘occurrence’ and ‘Australia’. Reference lists of relevant publications were also screened to identify early records not captured in search results.

**FIGURE 1:**
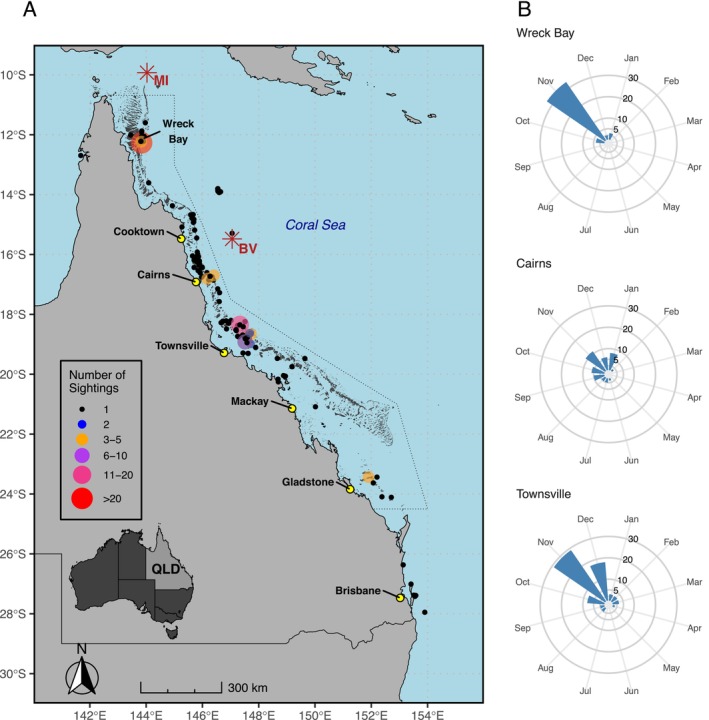
Historical sightings of whale sharks ( *Rhincodon typus* ) along the East Coast of Australia. (A) Sightings records are shown as circles, with size and colour differentiating the number of sighted whale sharks recorded in sightings databases between 2007 and 2018. Star‐shaped locations depict notable evidence of whale shark clusters mentioned in scientific literature at Murray Island (MI) (Simmons and Marsh 1986; Whitley 1965; Wolfson 1986) and Bougainville Reef (BV) (Hampton and Gunn 1998). For the three apparent hotspots that emerged from this work (Wreck Bay, Cairns, Townsville), (B) illustrates the monthly distribution of sightings in these regions. The Great Barrier Reef Marine Park boundary is shown as a dotted line. QLD, Queensland.

A review of published literature revealed that the first scientific evidence of whale sharks on the east coast of Australia emerged in the 1960s, summarised in Wolfson (1986). A number of single whale sharks were recorded along the New South Wales coast between 1936 and 1965 (Whitley 1965; Wolfson 1986). Whitley (1965) also mentioned single sightings off Townsville in 1955 and 1956, and, more significantly, ‘a group of about 30 whale sharks’ near Murray Island at the northern extent of the GBR ‘early in 1963’. In November 1983, a group of four whale sharks was also observed near Murray Island (Simmons and Marsh 1986). Together, these observations provide the first documented indication that whale sharks may aggregate on the east coast of Australia (Figure 1A: ‘MI’). In November 1985, aerial surveys conducted for dugongs ( *Dugong dugong* ) recorded nine whale sharks on the mid to outer shelf near latitude 12°S; however, all sightings involved solitary individuals (Marsh 1990). Later, in October–November 1991, a ‘large number’ of whale sharks was reported at Bougainville Reef in the Coral Sea off Cooktown (Figure 1A: ‘BV’), associated with aggregations of yellowfin tuna ( *Thunnus albacares* ) and bigeye tuna ( *T. obesus* ) feeding on lanternfish spawn (Hampton and Gunn 1998). Similarly, Wolanski and Hammer (1988) noted that whale sharks have been observed swimming and feeding on plankton slicks associated with upwelling in the centre of passages in the Ribbon Reefs in the northern GBR. While the timing of the latter observation is unclear, all other reports occurred in the months of October and November and were reported in association with other megafauna and/or abundance of plankton, suggesting that the northern GBR area is productive at this time of the year and potentially suitable for whale shark aggregations.

**FIGURE A2:**
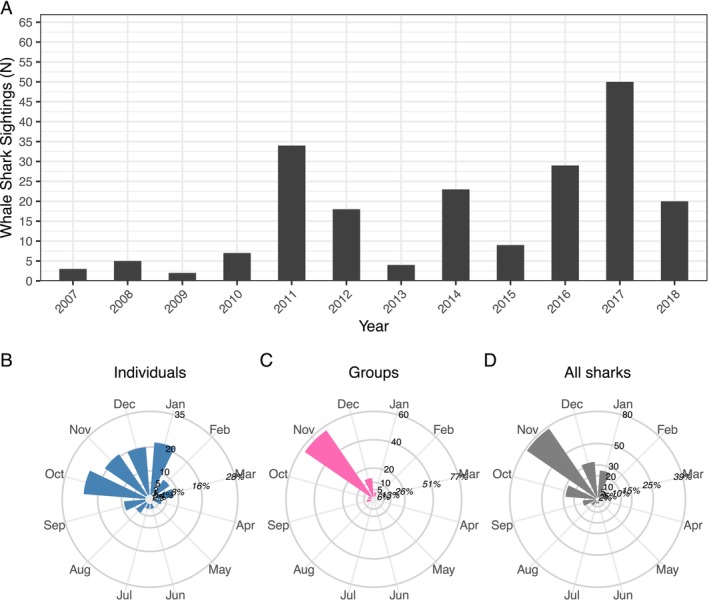
Whale shark ( *Rhincodon typus* ) sightings recorded on the east coast of Australia from public data bases, social media and anecdotal reports, excluding duplicated records. (A) Annual whale shark sightings from 2007 to 2018, and monthly distribution of sightings for individual sharks, groups and all sharks in (B), (C) and (D), respectively.

With the implementation of public sightings reporting networks in the early 2000s, the number of whale shark sightings reported on the east coast of Australia has steadily increased. Between 2007 and 2018, 204 whale sharks were recorded from 136 encounter reports, with annual numbers reported rising after 2010 (Figure A2A). A lower number of sightings reported before 2011 likely reflects the early stages of these networks, as public awareness of the reporting tools was developing. For the most part, sightings were sporadic along the Queensland coast and mostly individual whale sharks were reported (Figure 1, Figure A2). While sharks were spotted all year round except for May, nearly three‐quarters (71%) of single individuals were spotted between October and January (Figure A2). Only 10 of the encounter reports consisted of two or more whale sharks; however, those groups amounted to nearly 40% (*n* = 78) of all spotted sharks. Importantly, grouped whale sharks were exclusively recorded between September and January, with 90% of these sightings in November and December, and 73% in November alone (Figure A2C). While some sightings occurred in southern Queensland, including off Brisbane, and Gladstone at the southernmost extent of the GBR, the majority were reported between the Townsville region in the central GBR and the far northern GBR around Wreck Bay (Figure 1). The bulk of the reports were concentrated in the Townsville and Cairns regions, likely due to higher human population density and the Cairns region being the busiest reef tourism hub in Queensland (GBRMPA 2025), resulting in more people engaging in water activities compared to more remote areas further north or south.

**FIGURE 2:**
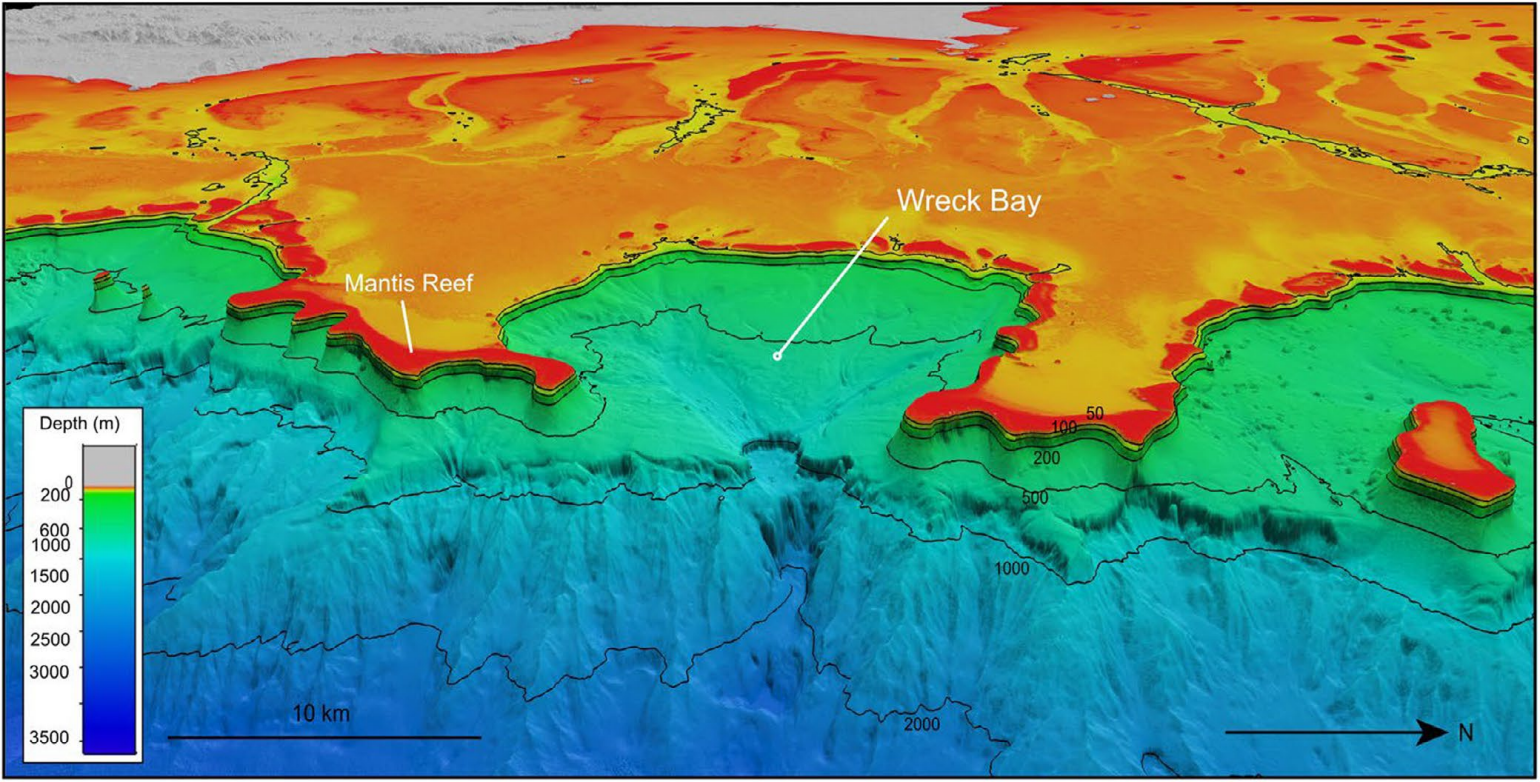
Westerly‐looking 3D view of Wreck Bay, Great Barrier Reef, Australia. Bathymetry data courtesy of the Schmidt Ocean Institute and Australian Hydrographic Office. Vertical exaggeration ×3.

Groups of whale sharks were observed in three key areas—Townsville in the central GBR, and Cairns and Wreck Bay in the northern GBR (Figure 1). The only other site where more than one individual was recorded was off Gladstone, where three whale sharks were sighted in 2016, marking the southernmost record of grouped whale sharks. Between 2014 and 2017, five multi‐individual encounters were recorded off Townsville, with group sizes ranging from two to 20 individuals. One of these reports noted that a group of seven whale sharks was feeding on the surface alongside large schools of tuna. Cairns had two reports of three whale sharks occurring as a group, both in the month of November, in 2007 and in 2008. No other encounters of multiple whale sharks were reported around Cairns over the following 10 years. While whale shark encounters around Townsville and Cairns are spread out over a large area, the third hotspot had sightings of multiple whale sharks over the smaller area of Wreck Bay, located at the edge of the continental shelf in the remote far northern GBR (Figure 1). In November 2011, the largest confirmed report of a group of whale sharks on the east coast of Australia was reported from the southern border of Wreck Bay, inside Mantis Reef (cf. Figure 2), with a conservative estimate of 31 individuals (observed range of 12–50 animals from multiple reports of this encounter). It was also reported that the whale sharks were surface feeding on visibly dense patches of zooplankton. Another group of three sharks was observed in January 2018 also inside Mantis Reef. Within 5 days in October 2017, four individual whale sharks with different size estimates were observed in Wreck Bay, followed by another encounter 2 weeks later. It is likely that these encounters were of more than one individual, suggesting the presence of multiple sharks. However, to be conservative, they were considered as single individual sightings.

### 2.2 Physical Characteristics

Bathymetric features are strongly associated with whale shark aggregations, in particular where shallow depths are in close proximity to steep slopes into deep water (mesopelagic zone) (Copping et al. 2018). Such characteristics create favourable conditions for upwelling, resulting in increased primary productivity and zooplankton abundance, which in turn attracts filter feeding species such as whale sharks (Heyman et al. 2001; Reynolds et al. 2024; Robinson et al. 2013; Rowat et al. 2006). For example, in the Azores archipelago, whale sharks occur more frequently in areas with steep bathymetric slopes near seamounts, coinciding with increased productivity and feeding opportunities (Afonso et al. 2014). To identify potentially suitable areas for whale shark aggregations along the GBR, we visually inspected regionally compiled, high‐resolution (30 m) bathymetric grids obtained from Project 3D‐GBR (Beaman 2017). This dataset integrates multibeam data—cleaned of anomalous noise within Teledyne Caris HIPS & SIPS software (Teledyne Technologies Inc., CA, USA)—and LiDAR bathymetry data to provide a seamless 3D depth model for the GBR and Coral Sea. Further details and data are available on the AusSeabed Marine Data Portal (https://portal.ga.gov.au/persona/marine).

**FIGURE A3:**
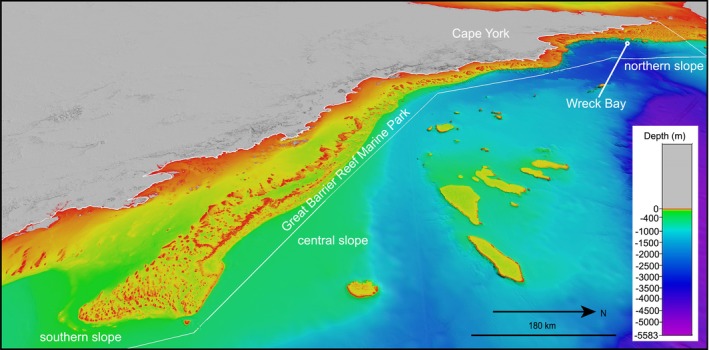
Westerly‐looking 3D view of the Great Barrier Reef, Australia. Bathymetry data courtesy of the Schmidt Ocean Institute and Australian Hydrographic Office.

The bathymetry and oceanographic conditions in the northern areas, where groups of whale sharks have repeatedly been recorded, are distinctly different from those in the central and southern GBR. Unlike the central and southern regions, which are in general characterised by shallow waters with gradual slopes into the deep waters of the Coral Sea, the remote northern GBR (north of Cairns) features steep bathymetric slopes that descend into deep waters of the adjacent Coral Sea (cf. Figure A3; Hopley and Smithers 2019). Such characteristics have been identified as key drivers of whale shark aggregations in other regions (Copping et al. 2018). Wreck Bay, where large groups of whale sharks have been repeatedly observed, has unique characteristics that distinguishes it from other nearby shelf areas both north and south. Wreck Bay consists of shallow, near‐surface coral reefs that form a bay‐like barrier, opening to the east (Figure 2). This formation provides physical protection from wind, waves and swell, facilitating calm conditions that likely retain and concentrate zooplankton, consequently ensuring a more consistent and predictable abundant food source for whale sharks. In contrast, upwelling‐induced zooplankton in the more open shelf areas to the north and south are likely to be more rapidly dispersed, making food less abundant and predictable. Wreck Bay has a funnel‐like shape, sloping from near‐surface coral reefs into increasingly deep waters. Its entrance reaches a depth of 1000 m, sloping further down to 2000 m and eventually to 4000 m in the Coral Sea basin (Figure 2). The northern GBR shelf region experiences coastal upwelling during the monsoon season (November–February), bringing nutrient‐rich deep water to the otherwise oligotrophic shallows (Berkelmans et al. 2010; Kingsford and Wolanski 2019; Sun et al. 2024; Wolanski et al. 1988). This aligns with previous findings suggesting that the occurrence of whale sharks and baleen whales on the east coast between 12°S and 14°S latitude during November was consistent with upwelling events that occur on the upper continental shelf of the GBR at that time of the year, and the resulting enrichment with nutrients and plankton (Marsh 1990; Wolanski and Hammer 1988). However, upwelling is not limited to the northern continental shelf regions. For example, the identified whale shark hotspots off the coast of Townville also coincide spatially and temporarily with intrusive upwelling, which facilitates the exchange of water between the shelf and the Coral Sea through channels—a phenomenon commonly observed in the central GBR (Benthuysen et al. 2016).

### 2.3 A Significant Piece of the Puzzle

**FIGURE 3:**
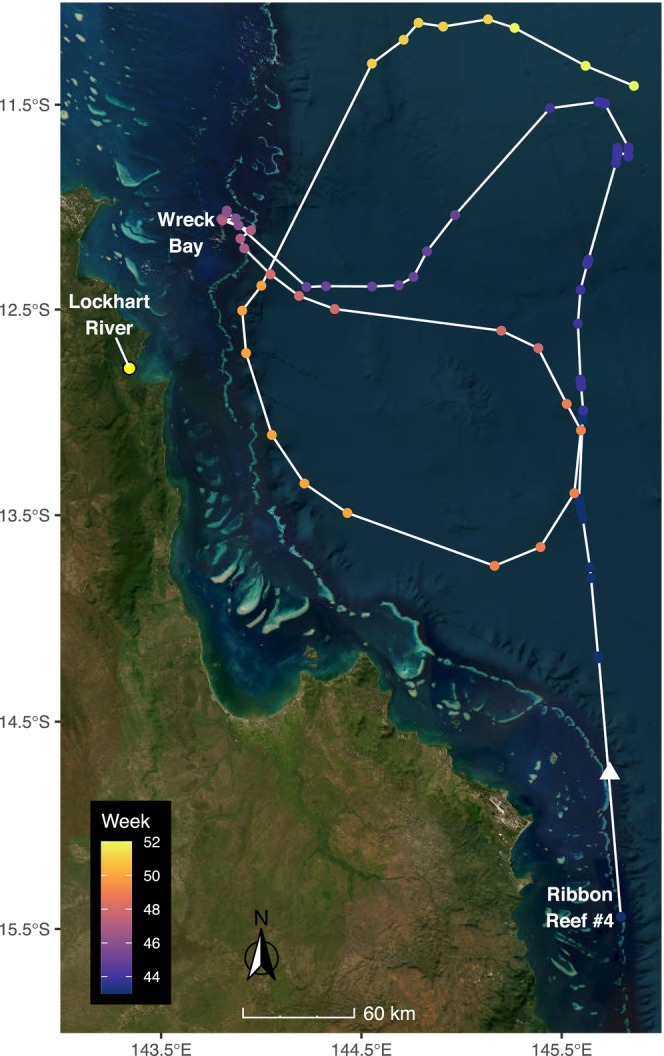
Satellite track of a whale shark ( *Rhincodon typus* ) opportunistically tagged at Ribbon Reef No. 4, off Cooktown, in October 2018. This individual moved to and resided in the Wreck Bay area for two weeks in November, confirming the projected location and timing that an aggregation is likely to occur. Satellite ‘World Imagery’ basemap provided by Esri, DigitalGlobe, GeoEye and other contributors, accessed using the ‘basemaps’ R package (Schwalb‐Willmann 2024).

While evidence was increasingly pointing towards Wreck Bay as the most suitable area to explore for a whale shark aggregation, the next piece of the puzzle was delivered by opportunistically equipping a whale shark at Ribbon Reef No. 4, off Cooktown (Figure 3) with a satellite transmitter (see below for satellite tagging procedures) in October 2018—the first satellite tracked whale shark on the east coast of Australia. Post‐tagging, this individual shark moved to Wreck Bay, where it remained for 2 weeks in November, further supporting the suspected location and timing for an aggregation to occur. Based on this suite of preliminary information, the Wreck Bay region in the far northern GBR during the months of November–January appeared to be the most likely site to find a whale shark aggregation.

## 3 Methods

An initial field expedition to Wreck Bay was undertaken in November 2019 to confirm the existence of the suspected aggregation. Three further voyages were conducted in 2021, 2023, and 2024 during the months of November/December to confirm that the site is an annual aggregation and to conduct further sampling. The expeditions included aerial surveys to find whale sharks, satellite tagging and photo ID to track shark movements and to characterise the demography of the aggregation (Figure A1). All in‐water data collection on whale sharks were performed on snorkel. Aerial surveys and in‐water data collection (satellite tagging, demographic data) were conducted on five consecutive days during each expedition (2019: November 15–19; 2021: December 05–09; 2023: November 29–December 03; and 2024: November 28–December 02).

All data preparation, statistical analyses, modelling and visualisations were performed using RStudio (Posit Team 2024), based on the statistical computing language R (Version 4.3.2, R Core Team 2024). A significance value α of 5% was used for statistical tests. Statistical values are presented as mean and standard deviations (SD), unless otherwise stated. In case of skewed data, median values were given with the interquartile range (IQR) representing the variation in the data. Maps were created using the ‘ggplot2’ (Wickham and Sievert 2016), ‘basemaps’ (Schwalb‐Willmann 2024), ‘ggspatial’ (Dunnington 2023) and ‘sf’ (Pebesma and Bivand 2023) packages.

### 3.1 Aerial Surveys

While the expedition vessel was at Wreck Bay, a single‐engine high‐wing aircraft (Cessna types 172, 182, and 210) with either one or two designated spotters was used to locate whale sharks by flying line‐transects or circular search patterns throughout the Wreck Bay area during daylight hours. Active spotting time was 3.5–4.0 h per day and ranged between 09:00 h and 16:00 h. Position and time of sighting were recorded for all sighted whale sharks. Once a shark was located, the plane circled the individual and communicated its location and details to the expedition vessel.

### 3.2 Tagging Procedures and Demographic Data Collection

With guidance of the spotter plane, a drone (DJI Mavic Pro Cine) was manoeuvred to and hovered above the whale shark to provide a visual reference and direct the tender with the tagging team to the shark's location. Continuous communication between the spotter plane and the drone operator was maintained to ensure safe separation between the tender and the whale shark. Individual whale sharks were identified by photographing their unique spot patterns behind the gills and above the pectoral fins and submitted to the *Sharkbook: Wildbook for Sharks* library (https://www.sharkbook.ai/). Each shark was assigned a unique ID (Table A1) and checked for matches in the database to identify potential connectivity with other sites (Arzoumanian et al. 2005; Norman et al. 2017). The size (total length TL in meters) was approximated using objects of known dimensions as reference points, for example, researcher in the water, or boat (Norman and Stevens 2007; Reynolds et al. 2024). Sex was determined visually by inspecting the pelvic fins for the presence of claspers (Norman and Stevens 2007; Pierce, Pardo, et al. 2021). Long and thick claspers that extend past the pelvic fins indicate calcification and thus maturity (Norman and Stevens 2007; Rohner et al. 2015). Female maturity was assigned based on size, assuming maturity at around 9–10 m (Norman and Stevens 2007; Pierce, Pardo, et al. 2021). To compare differences in total length between males and females, a non‐parametric Wilcoxon rank‐sum test was performed using the ‘rstatix’ package (Kassambara 2023), as the size data were not normally distributed (assessed using a Shapiro–Wilk test).

Sharks within the Wreck Bay aggregation site were equipped with satellite‐linked platform transmitter terminal (PTT) tags (Table A2) (Wildlife Computers Inc., Redmond, WA, USA)—either with smart position and temperature (SPOT) or data archiving and satellite transmitting (SPLASH) tags—and tracked using the Argos‐CLS satellite network (https://www.argos‐system.org/). SPOT tags provide near‐live tracking of horizontal geolocation data, while SPLASH tags also record depth time series data. SPOT (*n* = 15; models: SPOT‐196, 257) and SPLASH tags (*n* = 2; model: SPLASH10‐346) were attached to the first dorsal fin using custom‐made spring clamps, equipped with a set of spikes that grip and retain the tag on the fin (Gleiss et al. 2009; Norman et al. 2016).

Two whale sharks were also opportunistically tagged outside the aggregation site using tethered SPOT tags (model: SPOT‐253): one off Cooktown in 2018 (the preliminary tagged individual; Figure 3) and one off Cairns in November 2023 (Figure 7). Unlike the other individuals, which were tagged in Wreck Bay while snorkelling, these sharks were tagged directly from a boat. A handheld pole spear with a custom‐made tip applicator was used to intradermally insert a titanium dart anchor (Wildlife Computers Inc., Redmond, WA, USA) (Hammerschlag et al. 2011) above the first prominent longitudinal ridge, approximately centred below the first dorsal fin. Because they were not approached in the water, no sex or Photo ID data could be collected; therefore, they were excluded from the demographic analysis; however, they were included in movement analyses.

### 3.3 Movement Data Analysis

To analyse movement patterns and residency in the Wreck Bay aggregation, transmitted geolocation data from a total of 19 tagged whale sharks were included—17 tagged during three dedicated field expeditions to Wreck Bay (2019, 2021, and 2023), and the two opportunistically tagged sharks off Cooktown and Cairns in 2018 and 2023 (see Figure 7 for locations). One SPOT tag (PTT 178950) failed to transmit data and was excluded, resulting in 18 shark tracks considered for further analyses. All tracks were processed using the ‘aniMotum’ R package (Jonsen et al. 2023). A correlated random walk state space model (SSM), which incorporates Argos Kalman filtered error ellipses, was fitted to all tracks, applying a speed filter of 1.15 m s^−1^ to remove implausible locations (Cade et al. 2020; Guzman et al. 2022). To determine residency of whale sharks within the Wreck Bay aggregation, each SSM track was clustered into behavioural segments using the ‘segclust2d’ package (Patin et al. 2020). The behavioural states were determined based on longitudinal and latitudinal displacement or based on speed and turning angle (Patin et al. 2020). Considered for residency analysis were those parts of the tracks, that indicated area restricted search behaviour. Detailed information on the segmentation process is provided in Table A3.

Home range analysis was conducted to determine hotspot areas within the vicinity of the aggregation habitat, as described previously in Abrantes et al. (2024). Briefly, resident phases of the tracks were used to calculate autocorrelated kernel density estimates (AKDE) for each whale shark using the ‘ctmm’ package (Calabrese et al. 2016; Fleming et al. 2017). Twelve sharks had suitable tracks for home range estimation, including two multi‐year tracks (PTTs 178954, 252324). For the latter sharks, AKDE's were computed for each year they visited Wreck Bay. This resulted in a total of 15 track sections considered for home range estimation. The resulting individual AKDEs were used to estimate a population home range (i.e., PKDE). The 95%, 50% and 25% PKDE's were extracted to visualise home range, core use areas and high‐intensity core areas, respectively, including their 95% confidence intervals.

## 4 Results

### 4.1 Aerial Surveys

The number of whale sharks spotted during aerial surveys increased over the years, with 13 and 25 whale sharks recorded in 2019 and 2021, and 67 and 53 in 2023 and 2024, respectively. In total, 158 sharks were recorded over the course of the four expeditions. However, it is likely that some of these sightings were of the same individuals.

### 4.2 Demography

**FIGURE 4:**
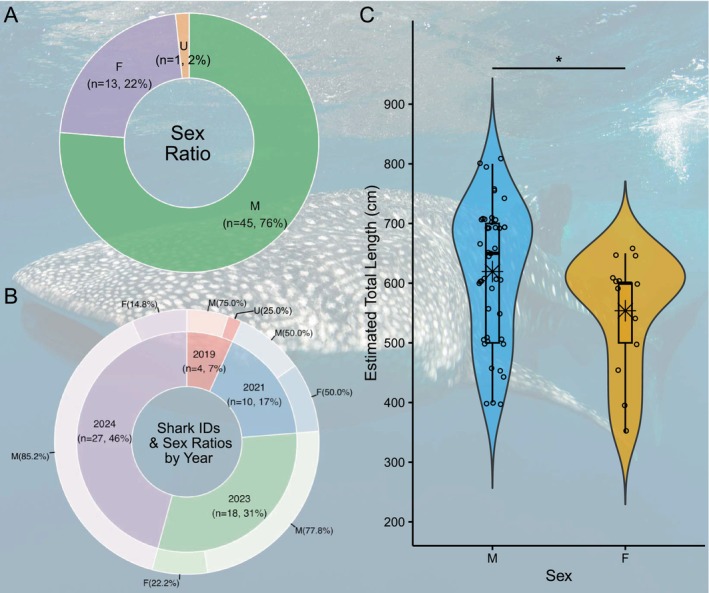
Demographics of the Wreck Bay whale shark ( *Rhincodon typus* ) aggregation. Overall sex ratios of the 59 individuals recorded by Photo ID are shown in (A), while (B) illustrates the proportion of whale sharks per year, and the sex ratios per expedition. Size distributions of males (*n* = 45) and females (*n* = 13) are shown in (C), with violin plots illustrating the density distribution of estimated total length. Boxes in the box‐and‐whisker plots depict the interquartile range (IQR, first and third quartile, Q1, Q3) and whiskers extend to 1.5× IQR from Q1 and Q3. The thick horizontal line within the box represents the median, while the star represents the mean. Raw data is shown as hollow circles. A Wilcoxon rank‐sum test confirmed significant size differences between the sexes (*W* = 184.5, *p* = 0.042, *n* = 48); indicated by an asterisk above the plots. F, female; M, male; U, undetermined.

A total of 59 individual whale sharks were recorded in‐water for photo‐identification and demographic analysis during the four expeditions to Wreck Bay (Table A1). The majority of these sharks were males, with an overall male to female ratio of 3.5:1 (males: *n* = 45, 76%; females: *n* = 13, 22%), while the sex of one individual was not determined (Figure 4A). Sex ratios varied over the years as the number of sharks identified increased (Figure 4B). Of the four sharks identified in 2019, three were males, while the sex ratio of the 10 photo ID'd sharks in 2021 was balanced. The sex ratios in 2023 and 2024 were 3.5:1 (males: *n* = 14, 77.8%; females: *n* = 4, 22.2%) and 5.8:1 (males: *n* = 23, 82.1%; females: *n* = 5, 17.9%), respectively. Estimated total length ranged from 3.5 to 8.0 m (Figure 4C). All sharks were immature, with four males classified as subadults based on clasper elongation without full calcification. Males were significantly larger (*W* = 184.5, *p* = 0.042, *n* = 48), with an average size of 6.2 ± 1.1 m (range: 4.0–8.0 m) compared to 5.5 ± 1.0 m (range: 3.5–6.5 m) for females. Photo ID confirmed that one shark that was documented in 2023 (GBR‐052) revisited Wreck Bay in 2024. None of the whale sharks had been identified elsewhere within the global Sharkbook database as of March 2025.

### 4.3 Residency and Movement Patterns

**FIGURE 5:**
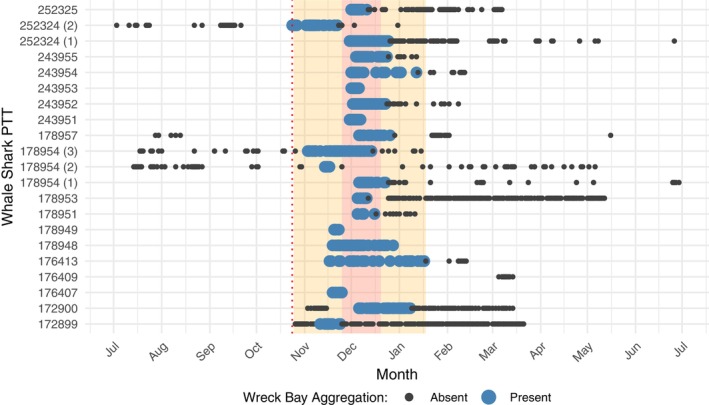
Whale shark ( *Rhincodon typus* ) residency based on the presence (large blue dots) and absence (black dots) in the vicinity of the Wreck Bay aggregation site, shown over a generic calendar year. The orange rectangle represents residency in Wreck Bay based on the earliest arrival and latest departure, while the red rectangle shows core aggregation period based on the median arrival and departure dates. The red dotted vertical line represents the earliest tag deployment (24 October). Numbers in parentheses after whale shark PTTs indicate the different aggregation seasons for multi‐year tracks.

Twelve tracked sharks (two females, eight males and two with undetermined sex) had suitable movement patterns (in terms of tracking duration and number of transmitted geolocations) to allow for a distinct separation of movement behaviours, including the departure from the Wreck Bay aggregation area (Table A3). The mean residence time in the general vicinity of the Wreck Bay area was approximately 3 weeks (21.6 ± 10.1 days; range: 7–43 days), during which time an average of 43.1 ± 35.5 geolocations (range: 7–145) were transmitted. The residence time is likely underestimated as it remains unclear how long the individuals had already been present in Wreck Bay before tagging. However, data from four sharks that were either tagged before reaching the aggregation area (*n* = 2) or tracked over multiple years after being tagged in Wreck Bay (*n* = 2) provide more accurate residency times. Based on track segmentation analysis of the two sharks tagged offshore from Cooktown and Cairns, one individual (PTT 172899, sex unknown, 5.0 m TL) remained in the Wreck Bay aggregation area for 14 days, while the other (PTT 172900, sex unknown, 5.1 m TL) stayed for about 1 month (34 days). For the two sharks tagged in Wreck Bay, a female (PTT 178954, 6.0 m TL) revisited the aggregation for two subsequent years, spending 11 days in the area during its first return in 2022 and 40 days in the second return in 2023. A male shark (PTT 252324, 7.0 m TL) tagged in 2023 spent approximately 1 month (30 days) in the aggregation area when it returned in the following year in 2024. Together, these four tracks suggest a general mean residence time of approximately 3 weeks (24.3 ± 10.9 days), similar to the estimated residence time from all 12 sharks. Acknowledging the relatively small sample size, the data collected so far suggest that the core aggregation period at Wreck Bay occurs between late November and late December, with the earliest arrivals at the end of October and the latest departures in mid‐January (Figure 5). Potential area‐restricted search behaviour (based on track segmentation analysis) predominantly occurred on the continental shelf area of the outer reefs, within approximately one degree latitude north and south of Wreck Bay, covering a latitudinal span of approximately 200 km.

**FIGURE 6:**
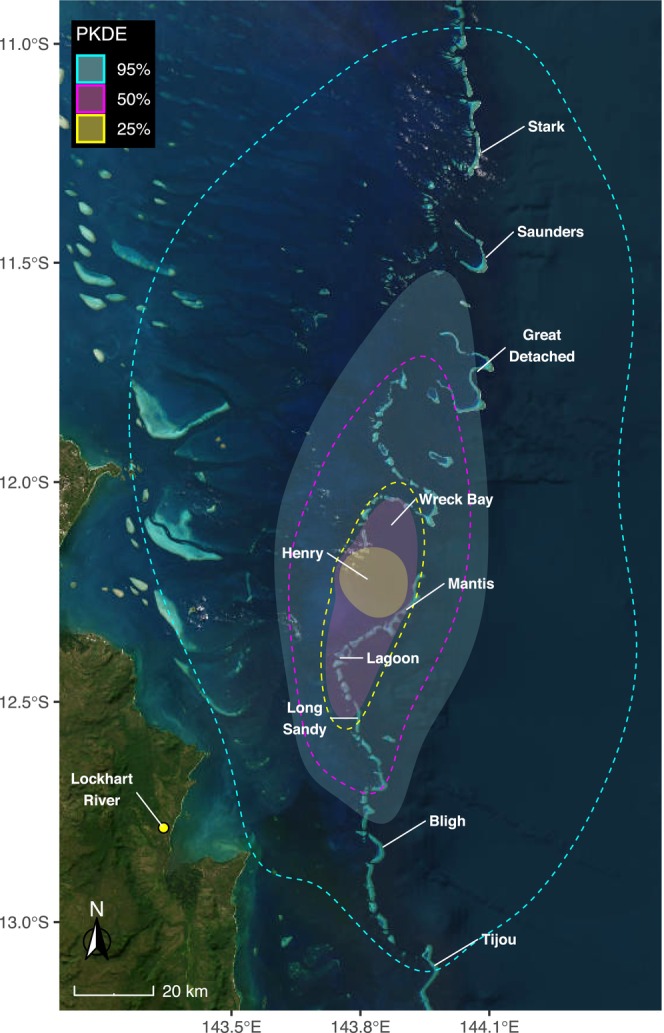
Population home range of aggregating whale sharks ( *Rhincodon typus* ) in the Wreck Bay area. Autocorrelated kernel density estimators (AKDE) were modelled for each shark's area‐restricted search behavioural segment of the tracks, and subsequently the populations' KDE (PKDE) was estimated. The 95% (turquoise), 50% (magenta) and 25% (yellow) PKDE's reflect the populations home range, core use areas and high‐intensity core areas while aggregating in Wreck Bay, respectively. Dashed lines represent the upper 95% confidence intervals for each PKDE. Annotations in white depict the locations and names of coral reefs. Satellite ‘World Imagery’ basemap provided by Esri, DigitalGlobe, GeoEye and other contributors, accessed using the ‘basemaps’ R package (Schwalb‐Willmann 2024).

Home range analysis of the overall tracked population suggests a home range area (95% PKDE) of 5336 km^2^ (17.9–24,411.1 km^2^ 95% CI), stretching over 140 km from the southern end of Saunder Reef to north of Bligh Reef (Figure 6, Table A4). The core use (50% PKDE) extends over 57 km from the northern end of Wreck Bay to Sandy Reef in the south and covers an area of 815.5 km^2^ (2.7–3730.9 km^2^ 95% CI). The 25% PKDE area is centred around Henry Reef, covering an area of 238 km^2^ (0.8–1090.0 km^2^ 95% CI).

**FIGURE 7:**
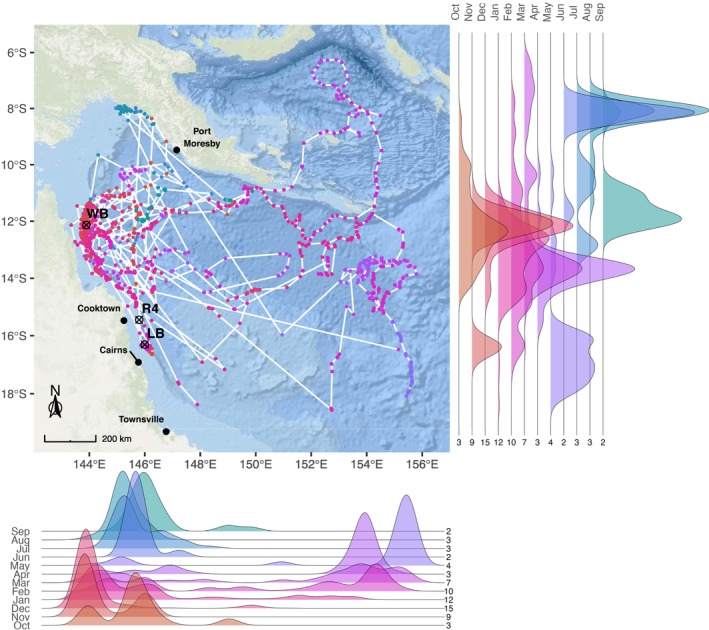
Spatiotemporal distribution of whale sharks ( *Rhincodon typus* ) (*n* = 18), satellite‐tracked between October 2018 and December 2024. Map shows the transmitted geolocations by month and paths of individual sharks connected by white lines, with density plots indicating monthly latitudinal and longitudinal distribution of geolocations and values indicating the number of individual sharks per month. Crossed circles indicate tagging locations: Wreck Bay (‘WB’), Ribbon Reef No. 4 (‘R4’) off Cooktown and Linden Bank (‘LB’) off Cairns. ‘Ocean Basemap’ data product provided by Esri, GEBCO, NOAA and other contributors.

Upon departing from Wreck Bay, whale sharks dispersed into the Coral Sea and beyond. This pattern was most apparent between January and May, when geolocations were spread across a broad spatial range in both latitude and longitude (Figure 7). Overall, the movements of tracked sharks spanned over 13° longitude (143°E to 156°E) and 12° latitude (18.6°S to 6.2°S). Distance travelled ranged from 19 km to 11,355 km track length per individual, with a median distance of 1463 km (2312 km IQR) (Table A2). Tag retention varied from 3 to 770 days, with an average of 144 ± 209.63 days at liberty (DAL, Table A2). Of the three female tagged sharks, one tag failed to transmit data (PTT 178950). The other two females exhibited considerably longer tracks compared to males (*n* = 13) and sharks with unknown sex (*n* = 3). One female (PTT 178957, 6.0 m TL) transmitted for 526 days, and the other (PTT 178954, 6.0 m TL) transmitted for 770 days. Male shark track durations ranged from 3 to 397 days, with an average of 78.5 ± 106 DAL, similarly to the sharks with unidentified sex, which averaged to 94.7 ± 77.1 and ranged from 6 to 146 days. Interestingly, both females visited the Gulf of Papua, while no male sharks moved to that area.

## 5 Discussion

Here, we describe the first whale shark aggregation site documented on the east coast of Australia and the broader southwest Pacific region east of Australia. Our investigative approach, layering multiple sources of information including insights from local experts and citizens' observations alongside knowledge from research at known whale shark aggregations, was paramount to identify and confirm the location of an aggregation site in this region. Through four targeted field expeditions, we can now confidently confirm a seasonal whale shark aggregation at Wreck Bay, situated at the far northern Great Barrier Reef.

To date, data suggest that the aggregation is dominated by juvenile males (75%, 4–8 m TL), which aligns with most known whale shark aggregations globally, where the percentage of males typically ranges from 62% to 97% and size distributions fall between 3 and 9 m (Araujo et al. 2022; Norman et al. 2017; Pierce, Pardo, et al. 2021). Sex ratios at whale shark hotspots closest to Wreck Bay are similarly male‐biased, with ~80% males at Ningaloo Reef (Western Australia) and ~65% to exclusively male individuals reported across Indonesian aggregations (Araujo et al. 2022; Meyers et al. 2020; Norman et al. 2017; Yasir et al. 2024). Notable exceptions from the predominant male bias include aggregations in Shib Habil in the Red Sea, Saudi Arabia (Berumen et al. 2014; Cochran et al. 2016), and St. Helena in the Atlantic Ocean, where sex ratios are more balanced, with the latter dominated by mature sharks and with evidence of mating (Perry et al. 2020). Darwin Island, in the Galápagos, remains the only documented hotspot with a predominance of adult females (Hearn et al. 2016, 2021). Given that demographic data are currently available for only 59 sharks in Far North Queensland, and that sex ratios varied across years, further data collection is required to comprehensively characterise the demography of the Wreck Bay whale shark aggregation.

The mean residency period of 3 weeks in Wreck Bay is consistent with other aggregations, where residencies typically range from 19 days at St. Helena (Perry et al. 2020) to around 1 month at Ningaloo Reef (Holmberg et al. 2009) and Holbox/Isla Mujeres at the Caribbean coast of Mexico (Hueter et al. 2013), and 2 months in Baja California (Ramírez‐Macías et al. 2012). However, the number of tagged sharks with a full year of data or more, needed to confirm site fidelity over multiple years, remains limited. In the present study, three individuals, two of whom were females, transmitted data for more than 1 year, providing some insight into potential multi‐year residency patterns and site fidelity. Two of these individuals revisited Wreck Bay in subsequent years, while the third (PTT 178957), a female tagged in 2021, exhibited sporadic and inconsistent data transmission with gaps of several months, with only 84 recorded locations over 500 days. Its last known position, recorded in May 2023, was in the Coral Sea off the northern Ribbon Reefs, about 100 NM south of Wreck Bay. While transience is a common trait in whale sharks across global aggregations (Pierce, Pardo, et al. 2021), the single resighted shark in Wreck Bay is considerably lower than the 36% resightings on average reported at other aggregations (Norman et al. 2017). In Wreck Bay, logistical constraints due to the area's remoteness limited sampling effort to only 5 days per season in 4 years since 2019. In contrast, at Ningaloo Reef, where 40% of identified animals were sighted over two or more calendar years, fieldwork has been ongoing for decades and occurs throughout the entire aggregation season (March–July), facilitated by research teams and citizen science initiatives (Meekan et al. 2006; Norman et al. 2016, 2017). Since 1986, over 2300 individuals have been recorded in the Sharkbook database for Ningaloo Reef. Similarly, in the Philippines, where the resighting rate is 35%, whale sharks are observed year‐round (Araujo et al. 2022), with over 800 individuals added to Sharkbook since 1998. Given our restricted sampling effort, it is possible that some previously identified individuals were in the area but remained undetected in subsequent years. Lack of resightings could also be influenced by the aggregation being distributed across a broad area, as indicated by home range analysis, and/or sharks spending limited time at the surface during the day.

Daytime surface feeding was observed occasionally, and several individuals were also seen ram‐feeding on dense zooplankton patches at night (from sunset to late into the night). Whale sharks show high plasticity of feeding behaviours, depending on the local food availability, ranging from passive, active and stationary feeding (Rohner and Prebble 2021) on the surface and in the water column at depth (Rohner et al. 2013), and benthic foraging (D'Antonio et al. 2024; Whitehead and Gayford 2023). Most commonly observed at coastal aggregations is active surface feeding during the day (Motta et al. 2010; Rohner and Prebble 2021), although observation bias may play a role in this (Rohner and Prebble 2021). Nocturnal surface feeding on high‐density plankton patches, as observed on multiple occasions in Wreck Bay, has, to our knowledge, previously only been regularly documented (under natural conditions) at Ningaloo Reef, where similar surface ram‐feeding behaviour was reported to occur during sunset (Gleiss et al. 2013; Taylor 2007). While further investigation is needed to quantify feeding patterns, these observations suggest that nocturnal foraging may be the predominant feeding behaviour in Wreck Bay. Preliminary analysis of the zooplankton community associated with the observed nocturnal feeding events in Wreck Bay showed a dominance of euphausiids (krill), along with planktonic tunicates, amphipods, crab megalopa, calanoid copepods and medusozoans also present. The taxa observed within the dense plankton patches generally overlap with those reported at other aggregation sites (Rohner and Prebble 2021), with krill in particular also documented as a key prey item at Ningaloo Reef (Taylor 2007). However, further data and detailed trophic analyses (e.g., stable isotope analysis) are needed to determine the specific diet of whale sharks in Wreck Bay.

Whale sharks are not the only marine megafauna that utilise Wreck Bay during summer months, with black marlin ( *Istiompax indica* ) known to routinely migrate north from the Ribbon Reef group of Cairns, up to Wreck Bay during this period (Domeier and Speare 2012; Williams et al. 2017). The spatiotemporal overlap of black marlin and whale sharks is not restricted to the Wreck Bay location, with these species co‐occurring in other parts of the world including Ningaloo Reef in Western Australia and Bazaruto Island in Mozambique (Pepperell et al. 2011; Rohner et al. 2014; Rohner, Bealey, et al. 2021; Wilson et al. 2001). Given that these species are among the most highly mobile on the planet, it suggests that the biophysical attributes of these sites offer an important ecological niche that can support the needs of migratory species that forage at different ends of the food web (Domeier and Speare 2012; Williams et al. 2017). During our field surveys, we also observed manta rays (*Mubula alfredi* and *M. birostris* ) and Omura's whales ( *Balaenoptera omurai* ) within Wreck Bay. Further work to understand whether the timing and occurrence of black marlin and other co‐occurring megafauna species are associated with specific physical factors would help to identify other whale shark aggregations as well as the ecological value of these sites.

Extensive whale shark movements have rarely been recorded, likely due to short satellite tag deployments and tag shedding (Hearn et al. 2021). In the current study, a female whale shark travelled at least 11,355 km over a period of over 2 years (770 days), representing one of the longest tag deployments and distances moved. Despite the long track distances indicating wide dispersal capabilities, most of the tracked whale sharks in this study remained within the Coral Sea region, except for two individuals that moved into the Solomon Sea. Notable transoceanic movements include a 7.1‐m whale shark of unknown sex that travelled nearly 13,000 km from the Sea of Cortez into the northwestern Pacific over a period of 3 years (Eckert and Stewart 2001) and a 7‐m female that travelled > 20,000 km from Panama to the Mariana Trench in 841 days (Guzman et al. 2018). However, the accuracy of these tracks has been questioned due to the lack of depth data and inconsistencies in location fixes (Hearn et al. 2021). The longest verified movement to date was recorded from an 8 m female fitted with a fin‐mounted SPOT tag, travelling > 40,000 km over 4 years, mostly within the Gulf of Mexico (Daye et al. 2024).

In conclusion, we discovered the first whale shark aggregation on the east coast of Australia, which occurs annually at Wreck Bay in the remote northern Great Barrier Reef during the months of November and December. This is a first step to filling a significant gap in whale shark knowledge in the Coral Sea. Unlike most whale shark studies, which typically commence at established aggregation sites, our research took the opposite approach—we set out to investigate whale sharks on Australia's east coast without knowing where to start. This ‘needle in the haystack’ challenge required an innovative investigative strategy, combining local and expert knowledge, citizen science, and an understanding of what is driving whale shark movements and aggregations in other areas to pinpoint a previously unknown aggregation. This approach demonstrates how aggregations can be identified in data‐poor regions and could be applied to other species or locations where critical habitats remain undocumented. While public databases have proven to be valuable resources, unfortunately we found that many whale shark encounters remain unreported. The identification of this critical habitat has important implications for the Great Barrier Reef Marine Park and Coral Sea Marine Park. Currently, whale sharks are not a priority for management in this region, however the data presented here highlights its importance for the species and emphasises the need to assess potential threats to whale sharks in their newly identified essential habitat. Nevertheless, beyond the GBR, our findings will contribute valuable data for future conservation assessments for this Endangered species (Pierce and Norman 2016). Importantly, our findings indicate that Wreck Bay may not be the only aggregation site along the east coast of Australia. Sightings data highlighted other locations where groups of whale sharks have been repeatedly observed (i.e., off Townsville, Cairns), suggesting the possibility of additional, yet undocumented, aggregations. Furthermore, our tracking data revealed additional areas of interest where no prior sightings have been reported, such as the Gulf of Papua, underscoring the potential for undiscovered aggregation sites across the broader southwest Pacific region. Building species distribution models to identify potential suitable and essential habitats to target further sites and expand research efforts, would be valuable steps towards improving our understanding of whale shark distribution, ecology, and aggregation dynamics in the Coral Sea and beyond.

## Author Contributions

**Ingo B. Miller:** conceptualization (equal), data curation (lead), formal analysis (lead), investigation (equal), methodology (lead), project administration (equal), visualization (lead), writing – original draft (lead), writing – review and editing (lead). **Richard Fitzpatrick:** conceptualization (equal), funding acquisition (equal), data curation (equal) project administration (equal), resources (equal), writing – review and editing (equal). **Kátya G. Abrantes:** conceptualization (equal), funding acquisition (equal), project administration (equal), writing – review and editing (equal). **Bradley Norman:** conceptualization (equal), investigation (equal), writing – review and editing (equal). **Simon J. Pierce:** conceptualization (equal), data curation (equal), investigation (equal), writing – review and editing (equal). **Mark V. Erdmann:** funding acquisition (equal), investigation (equal), resources (equal), writing – review and editing (equal). **Lisa A. Hoopes:** funding acquisition (equal), investigation (equal), resources (equal), writing – review and editing (equal). **Christine Dudgeon:** conceptualization (equal), writing – review and editing (equal). **Matthew D. Dunbabin:** investigation (equal), methodology (equal), resources (equal), writing – review and editing (equal). **Alistair D. M. Dove:** conceptualization (equal), funding acquisition (equal), investigation (equal), methodology (equal), resources (equal), writing – review and editing (equal). **Robin J. Beaman:** data curation (equal), investigation (equal), resources (supporting), visualization (supporting), writing – review and editing (equal). **Samantha D. Reynolds:** investigation (equal), writing – review and editing (equal). **Christopher Rohner:** investigation (equal), writing – review and editing (equal). **Samuel M. Williams:** writing – review and editing (equal). **David Paton:** resources (supporting), writing – review and editing (equal). **Sonny Lewis:** investigation (equal), writing – review and editing (equal). **Adam Barnett:** conceptualization (lead), data curation (equal), funding acquisition (lead), investigation (equal), project administration (lead), resources (equal), writing – review and editing (equal).

## Permits and Ethics

Research was conducted under permits G18/39348.1, G21/144109.1 and G22/46908.1. All work conducted was approved by James Cook University Animals Ethics Committee (A2864).

## Conflicts of Interest

The authors declare no conflicts of interest.

## Data Availability

Sightings data as well as tracking data used for analysis are available on the Zenodo data repository: https://doi.org/10.5281/zenodo.15048664. Near‐live tracks can be viewed at https://biotracker.tv/. Photo IDs were uploaded to *Sharkbook: Wildbook for whale sharks*: https://www.sharkbook.ai/ and links provided in Table A1.

## Appendix A

**TABLE A1:** Photo IDs of Wreck Bay whale sharks ( *Rhincodon typus* ) submitted to *Sharkbook: Wildbook for Sharks* (https://www.sharkbook.ai).

Shark ID	Sex	TL	Expedition year	PTT	Sharkbook encounter link
GBR‐001	M	700	2021	178951	9cf2da54‐28d2‐448b‐acfa‐ea285aec362c
GBR‐002	M	550	2021		8eae9454‐2301‐435b‐96b4‐dfd4c3f6f6c5
GBR‐003	F	500	2021		c9834549‐ac87‐4903‐ae74‐6f8f434969d7
GBR‐004	M	650	2021		2c448119‐5cfe‐4476‐8b73‐246c01be5e24
GBR‐005	M	700	2021		9968c8a9‐b0bc‐45ef‐8940‐757a0db4cbd4
GBR‐006	F	600	2021	178954	000588bf‐460f‐49c7‐99b9‐fe9a0801640f
GBR‐007	F	600	2021	178957	83bb78b7‐8d13‐41e5‐b7c3‐56061ba8ecee
GBR‐008	F	600	2021		a5c08b42‐90d2‐4ef6‐b4a0‐3214fc51d133
GBR‐009	M	600	2021	178953	46c4f0dc‐6306‐4b1d‐bd97‐33d6c31f8126
GBR‐010	F	550	2021	178950	a9697a0e‐5837‐4673‐b299‐1dcdeceb5215
GBR‐044	F	600	2023		63921607‐6cc3‐4123‐9b7e‐38f6f0651642
GBR‐045	F	450	2023		270a7a9a‐c98e‐425f‐967c‐24439723414c
GBR‐046	M	700	2023	243951	219a61d9‐c57f‐4319‐a06f‐c2854c681e2a
GBR‐047	M	700	2023	252324	5803be05‐89e5‐49b8‐a917‐e23ad069c9c0
GBR‐048	M	550	2023		0350e4b7‐2d90‐4aba‐a6c1‐817b981cdeff
GBR‐049	M	600	2023	252325	40ffbc23‐2cd3‐4979‐854e‐5574413257f3
GBR‐050	M	600	2023	243953	06c0fccf‐d4c9‐4c37‐ab8b‐2127332a1a59
GBR‐051	M	800	2023	243952	4b093b96‐e374‐4071‐a16a‐6e1a100f3f77
GBR‐052	M	450	2023		aee3c3e3‐69ed‐4e41‐8e06‐4d30c483f317
GBR‐053	M	700	2023	176409	d7ffb68e‐1196‐41de‐a54e‐42a495a0101b
GBR‐054	M	450	2023		2307868c‐6d50‐48b6‐95ee‐a0a876fe81f7
GBR‐055	F	650	2023		0af57755‐623c‐4eb8‐8e6d‐8c3c9999a108
GBR‐056	M	675	2023	243954	3217982d‐c99c‐4b0b‐a0f7‐0c2a11a7bddd
GBR‐057	F	650	2023		588c7359‐2d45‐418c‐9eb2‐449f622495a3
GBR‐058	M	600	2023		380dc23f‐082c‐4411‐82b4‐fe85f299dec0
GBR‐059	M	650	2023	243955	f218f87b‐236c‐461a‐9f46‐22c28de20dc6
GBR‐060	M	400	2023		3283160e‐b863‐4a60‐b008‐0bb405b1432e
GBR‐061	M	400	2023		d568e7df‐3664‐4ffe‐af25‐7ab76858336a
GBR‐066	M	400	2019	176413	2a696b10‐69a7‐43c7‐945b‐dd937884b615
GBR‐067	U	400	2019	176407	b5c2190e‐d2f0‐4aa1‐93d8‐f18c96b252b3
GBR‐068	M	750	2019	178948	c85aa324‐012b‐42df‐9d93‐953b4de5e787
GBR‐069	M	700	2019	178949	bee933e8‐d64b‐4d95‐8556‐db45c182c71a
GBR‐070	M	650	2024		42013657‐cc6d‐458e‐8261‐80663a12bc99
GBR‐011	F	650	2024		f7ad700e‐9451‐48f8‐a432‐9d5a5eda574e
GBR‐012	M	700	2024		a27859fe‐7e44‐4a2f‐b63a‐75228af397cf
GBR‐013	M	750	2024		9749edd8‐6680‐4313‐84de‐e3ef0794e9ca
GBR‐014	F	400	2024		bb46a7e1‐de88‐4207‐b924‐99da76346faa
GBR‐015	M	700	2024		1d4b768d‐eb3c‐4576‐b97f‐aead6f2d3357
GBR‐016	M	500	2024		8b3ac1f7‐93c0‐4db6‐8d11‐d4677279a7b5
GBR‐017	M	800	2024		a754f8b0‐98fb‐4b29‐a302‐c44036ebf112
GBR‐018	M	700	2024		43c74038‐d691‐4f28‐85ff‐65b3f1aae0ae
GBR‐019	M	750	2024		7b1e4b87‐4a7b‐4598‐9b6a‐f2b51bc2020d
GBR‐020	F	600	2024		19c9da71‐0f84‐48aa‐98aa‐c1b647ae39f5
GBR‐021	M	600	2024		8fa5eec0‐cabb‐45da‐99e9‐cb12db42a6c3
GBR‐023	M	650	2024		617a8fb0‐bf86‐4847‐8c72‐a47b4fab57c9
GBR‐024	M	450	2024		285aa49d‐337d‐4817‐9132‐87fcbed12e58
GBR‐025	M	500	2024		5ed483c0‐f15d‐46c1‐868b‐649a53271b2a
GBR‐026	M	500	2024		1865e936‐67d5‐48da‐9dee‐ae81bc0fc42e
GBR‐027	M	700	2024		2fbff285‐4245‐4c96‐939d‐5abab875aefc
GBR‐028	M	800	2024		2d345183‐6054‐4d1f‐901a‐01bb6b09c515
GBR‐029	M	500	2024		a3312908‐56e7‐4a49‐8284‐70e5097b960e
GBR‐030	M	700	2024		841a944c‐f9e2‐4acd‐8b41‐3cbc67d654bd
GBR‐031	M	700	2024		31d73cc9‐2125‐4180‐9899‐de682e46b3a3
GBR‐032	M	600	2024		b2b57440‐db1a‐4dbe‐a647‐c8d2f1a403de
GBR‐033	M	600	2024		37fc2977‐91c1‐4f65‐94c9‐370a5a37d001
GBR‐034	M	500	2024		9e8d7286‐941f‐4744‐8413‐fc044a5b2f87
GBR‐035	M	700	2024		b9124650‐d9d8‐40cc‐8010‐939b56d35abc
GBR‐036	F	350	2024		c2034778‐7ae1‐4265‐a9eb‐7d5b9d013669
GBR‐037	M	500	2024		ec557c0b‐9113‐49d7‐91ec‐5cb17146b73a

*Note:* For individuals that were also satellite tagged, the corresponding Platform Transmitter Terminal (PTT) ID is provided.

**TABLE A2:** Meta data of tagged whale sharks on the east coast of Australia, including their respective number of locations transmitted, days at liberty (DAL) and track distance in km.

#	PTT	Date	Sex	Size	Tag	Mounting method	Locations	DAL	Distance km
1	172899	2018‐10‐24	U	500	SPOT‐253	Tether	185	146	3670
2	176413	2019‐11‐17	M	400	SPOT‐257	Fin Clamp	81	88	2672
3	176407	2019‐11‐18	U	400	SPOT‐257	Fin Clamp	9	6	18
4	178948	2019‐11‐19	M	750	SPOT‐257	Fin Clamp	73	39	255
5	178949	2019‐11‐19	M	700	SPOT‐257	Fin Clamp	11	3	20
6	178951	2021‐12‐05	M	700	SPOT‐257	Fin Clamp	29	37	865
7	178953	2021‐12‐05	M	600	SPOT‐257	Fin Clamp	461	158	4298
8	178957	2021‐12‐06	F	600	SPOT‐257	Fin Clamp	84	526	1934
9	178954	2021‐12‐06	F	600	SPOT‐257	Fin Clamp	333	770	11,355
10	172900	2023‐11‐03	U	510	SPOT‐253	Tether	235	132	2638
11	243951	2023‐11‐30	M	700	SPOT‐196	Fin Clamp	57	7	143
12	252324	2023‐11‐30	M	700	SPLASH10‐346	Fin Clamp	509	397	6067
13	243953	2023‐12‐01	M	600	SPOT‐196	Fin Clamp	22	5	76
14	243952	2023‐12‐01	M	800	SPOT‐196	Fin Clamp	126	68	1843
15	252325	2023‐12‐01	M	600	SPLASH10‐346	Fin Clamp	280	97	1538
16	243954	2023‐12‐01	M	650	SPOT‐196	Fin Clamp	78	73	643
17	176409	2023‐12‐02	M	700	SPOT‐253	Fin Clamp	28	9	1388
18	243955	2023‐12‐04	M	650	SPOT‐196	Fin Clamp	65	39	770
19	178950^a^	2021‐12‐05	F	550	SPOT‐253	Fin Clamp	1	0^a^	

Abbreviations: PTT, platform transmitter terminal; U, undetermined.

^a^ Tag failed—excluded from analysis; only used for demographic analysis.

**TABLE A3:** Details on parameters set in the clustering process for determination of shifts in behaviour in whale shark ( *Rhincodon typus* ) tracking data using the ‘segclust2d’ R package (Patin et al. 2020).

PTT	Covariates	lmin	Clusters	Min date; leaving date	Days within WB	No. locations within WB
172899	Speed (smoothed), turning angle	10	3	11/11/2018* 25/11/2018	14	10
172900	Speed (smoothed), turning angle	10	4	06/12/2023* 09/01/2024	34	63
176407				19/11/2019 Has not left WB	6	9
176409				First location away from WB and no area‐restricted search behaviour obvious		
176413	X, Y	5	5	17/11/2019 18/01/2020	17	32
178948				19/11/2019 Has not left WB	39	63
178949				20/11/2019 Has not left WB	3	10
178951	X, Y	5	3	05/12/2021 17/12/2021	13	11
178953	X, Y	10	5	05/12/2021 12/12/2021	7	14
178954	X, Y	5	5	Year 1: 06/12/2021 25/12/2021	19	23
				Year 2: 14/11/2022* 25/11/2022	11	7
				Year 3: 03/11/2023* 25/12/2023	40	60
178957	X, Y	5	5	06/12/2021 29/12/2021	23	17
243951				30/11/2023 Has not left WB	7	53
243952	X, Y	5	3	02/12/2023 2/12/2023	22	76
243953				01/12/2023 Has not left WB	5	18
243954	X, Y	5	3	01/12/2023 13/01/2024	43	45
243955	X, Y	5	3	04/12/2023 25/12/2023	21	45
252324	X, Y	10	3	Year 1: 30/11/2023 26/12/2023	27	145
				Year 2: 24/10/2024* 23/11/2024	30	55
252325	X, Y	10	5	01/12/2023 12/12/2023	11	49

*Note:* For each shark (PTT), the minimum date and date of leaving the Wreck Bay (WB) aggregation area with a clear shift in behaviour, the number of days spent within the aggregation area until leaving and the number of locations transmitted during that time are given. For animals and periods where a clear arrival date in the WB area is apparent, the minimum dates are marked with an asterisk.

Abbreviations: PTT, platform transmitter terminal; WB, Wreck Bay.

**TABLE A4:** Population kernel density estimates (PKDE) for whale sharks ( *Rhincodon typus* ) aggregating in the vicinity of Wreck Bay.

PKDE	Area (km^2^)	95% CI (km^2^)	
		Low	High
95% (home range)	5335.8	17.9	24,411.1
50% (core area)	815.5	2.7	3730.9
25% (high‐intensity core area)	238.3	0.8	1090.0
